# Professor Flint’s Introductory

**Published:** 1854-12

**Authors:** 


### PROFESSOR FLINT'S INTRODUCTORY.

The Introductory Lecture of Prof. Flint, at the opening of the Session for 1854–5 in the Medical Department of the University of Louisville, has been published at the request of his class. It is a scholarly, chaste, instructive discourse on topics connected with the medical profession suggested by the late tour of Prof. Flint in Europe. Speaking of the personal appearance of medical men in Europe as affected by years compared with the same class in our own country, Prof. F. remarks:—

"As regards the comparative duration of life, in Europe and America, of members of the medical profession, I am not prepared to form an opinion. Statistical data adequate to decide this point, so far as I know, are wanting. But that the exterual manifestations of accumalated years, as a general remark, are less conspicuous than with us, and the body, if not the powers of the mind, maintained in a better state of preservation in spite of the influences of declining age, I think cannot be doubted. The causes of this difference are probably to be found in the custom, in this country, to engage earlier in active professional duties, to incur sooner domestic responsibilities, and in a variety of ways to guard with less care against circumstances involving wear and tear of the physical and mental constitution."

In reference to the comparative cost of a medical education in Paris and in the schools of the United States, he makes the following statement:—

"It is not to be inferred from the fact of instruction in France being gratuitous, that a medical degree is obtained without expense. The medical student, during his four years of pupilage, is obliged to register his name every three months at the *bureau* of the medical faculty. For each of these *inscriptions*, as they are called, sixteen in number, a fee is required. He is also obliged to pay a fee for each of the five examinations to which he is subjected at different periods of his pupilage; for the examination and printing of his thesis, for the study of practical anatomy, and for his diploma, the aggregate amounting to about $250, a sum somewhat greater than is demanded for two courses of instruction at the most expensive of American colleges. I am led to make this statement because it appears to be a current belief that a medical education in Paris costs nothing, and the gratuitous instruction in France is sometimes cited as a precedent for the action of certain schools in this country, which have adopted a policy dictated by the supposition that their interests will be promoted by offering to students the temptation of low fees."

